# EDUCATION, GERONTOLOGY, AND MUSEUMS

**DOI:** 10.1093/geroni/igz038.1321

**Published:** 2019-11-08

**Authors:** Ray Williams

**Affiliations:** Blanton Museum of Art, Austin, Texas, United States

## Abstract

Many adult museum visitors welcome the invitation to make personal connections to works of art. Time for individual reflection and sharing with others may enable relationships to deepen and new insights to emerge. This presentation describes an approach to gallery teaching that honors the memories, associations, and emotions that visitors bring to their encounters with works of art. The approach has been particularly effective with groups of health care professionals, and as a reminder to docents of the powerful affective experiences that will naturally occur for some members of the public. Drawing on the experiences of collaborative networks of museum and medical educators, we outline the basis of rich opportunities for developing lifespan and gerontological educational projects in museums.

